# Frequency and Association of Stress Levels with Modes of Commuting Among Medical Students of a Developing Country

**DOI:** 10.21315/mjms2021.28.4.12

**Published:** 2021-08-26

**Authors:** Syed Muhammad Ashraf Jahangeer, Nimra Hasnain, Muhammad Taha Tariq, Ammara Jamil, Syeda Yamna Zia, Washma Amir

**Affiliations:** Department of Community Health Sciences, Dow Medical College, Dow University of Health Sciences, Karachi, Pakistan

**Keywords:** stress, transport, medical students, health, noise pollution, sleep

## Abstract

**Introduction:**

This study aimed at determining the frequency and association of stress levels with modes of transportation and was likely to reveal the contributing transportation-related factors for stress in medical students.

**Methods:**

This was a questionnaire-based, cross-sectional study that included undergraduate medical students of a public sector medical university in Karachi, Pakistan. A total of 573 students participated voluntarily, of which 300 were provided a manual questionnaire and 273 filled it online. The reliability of the questionnaire was assessed using Cronbach’s alpha at 0.791. The collected data were analysed using IBM SPSS Statistics for Windows, version 21.0.

**Results:**

Out of 573 participants, 99.3% *(n* = 298) of students filled the questionnaire manually, whereas 100% of students filled the questionnaire online. Almost two-thirds of students used university transport; more than 90% lived more than 5 km from the university and 56% had a traveling time of more than an hour. Approximately 15.4% of students reported physical trauma and some form of harassment. Sheldon Cohen’s stress scale surprisingly revealed 90% of students to be within the moderate-to-high stress category. Risk factors were associated with stress levels and significant associations were observed with noise exposure (*P* = 0.023) and sleep quality (*P* = 0.001). The most common reported stressors associated with commuting included overcrowding, long travel, and air and noise pollution. Noise pollution was the main predictor of stress among commuters.

**Conclusion:**

Poor transportation has adverse effects on health and academic performance. Administration in their respective jurisdictions is needed to investigate this matter to make commuting a routine rather than a hassle.

## Introduction

Stress has emerged as a major issue in today’s advanced world, affecting people from all walks of life. Various types and frequencies of stress are observed in different groups of people, revealing the contribution of different factors and predictors for it. Various studies on determining the factors related to stress have revealed several factors such as lifestyle, financial status, work-life balance and health status. Various studies conducted in developed countries have repeatedly reported transportation to be a significant stressor among the general population ranging from workers to students, wherein poor transportation affects the academic performance not only leading to lower test scores (1) but also adverse effects on psychological health (2). Various studies have investigated the association of travel time, duration, lack of predictability (3), and crowding with rising levels of stress and found a direct relationship between increased travel time and perceived poor sleep quality (4), low enthusiasm, low self-rated health and aggressive mood (5). Most of the studies have emphasised commute variability to be the strongest factor that influences commute strain (6).

Studies have particularly revealed the growing incidence of stress among students (7). A wide range of epidemiological studies and randomised control trials have been conducted to ascertain the prevalence, incidence, and causes of ominous levels of physical and psychological stress in medical students, as the association between stress in medical students and educational performance was well established (8). Comparison studies conducted between college and medical students evidently showed a higher rate of emotional exhaustion, depersonalisation, and burnout among the medical students (9). Considering the vast array of stress and its related factors, several factors among different groups of people have not been studied thoroughly. Evidence suggested the need for further investigations, including students from various educational backgrounds residing in different countries catering to different financial backgrounds to determine any additional factors that influence the level of mental distress (10). As the studies concerning stress levels and the potential predictors among medical students are scarce in developing countries, transportation-associated factors need to be found as a source of stress.

Stress-free commuting is a primary need for anyone who performs day-to-day multitasks and has the stress of academics such as medical students. Stress among medical students has been reported and linked with severe consequences (11). However, poor transportation contributing to stress among medical students has not been thoroughly studied in our setup. Therefore, our study aimed at determining the frequency and association of stress levels with modes of transportation and was likely to reveal the transportation-related factors contributing to stress in medical students. The main objective of the study was to assess the frequency of stress among medical students of Dow University of Health Sciences (DUHS), Karachi, Pakistan and its association with the transportation-associated stressor.

## Methods

Our study was conducted on students of Dow Medical College (DMC) in DUHS and the data were collected over 3 months. This was a cross-sectional study. The sample size was estimated using the OpenEpi software, an Open Source Epidemiologic Statistics for Public Health, version 3.01. With a prevalence of stress among medical students to be 31% and absolute precision to be 5%. The confidence level was 95%. The estimated sample size was 568 participants; however, we could collect data from 573 participants. The inclusion criteria set for the study included the following: all the medical students studying at DUHS and hailing from various ethnicities and residential areas with diverse financial backgrounds, including both men and women of any age group. In addition, students residing in the hostel were excluded from the study. Furthermore, students who did not give voluntary consent were excluded. The collected data were analysed using IBM SPSS Statistics for Windows, version 21.0 (2012, Armonk, NY: IBM Corp.).

### Data Collection

This was a questionnaire-based, cross-sectional study conducted on undergraduate medical students of the first to final years in DMC. Bachelor stream of medicine-surgery (MBBS) and dentistry (BDS) students were included in the study. Data were collected through both manual and online questionnaires. The detailed questionnaire was carefully designed under the direct supervision of an epidemiologist, an assistant professor at the community health department at DMC and was based on the available literature. The questionnaire comprised three sections. The first one is covering basic information, including demographic data, gender, and the mode of transport and associated factors, including travel duration, the reason for selection of that travel, stress-contributing factors in the mode of transport, and noise exposure. The second section is the Sheldon Cohen’s Perceived Stress Scale (PSS)-10 to determine the stress level of the participants, followed by a third section that collects information regarding the academic performance, sleep duration and quality, physical and mental health, trauma, physical or mental abuse, frequency of illness, and economic stability, all that could serve as contributing factors to the stress level of the participants. A pilot study using 15% of the sample size was conducted to evaluate the feasibility and appropriateness of the study operations. Cronbach’s alpha was used to determine the internal consistency and reliability of PSS in our population. Both reliability (α = 0.791) and validity of the PSS were similar to those reported in various studies (12, 13, 14).

The medium of the questionnaire was English. The systematic random sampling technique was used to reduce maximum possible bias, with no exclusion based on age, gender, year of study (except the first semester students, as they had less exposure to commuting to and from the university), course of study, and economic or religious background. Every measure has been considered to maintain the confidentiality of the participants. Participants were not identified by their names or email addresses, and no other identifying information was taken from them. Some general demographic information was collected, which cannot be used to trace back to them. Ethical standards were taken special care of, and no student was forced to participate in the study. Participation was on a completely voluntary basis, and data collection was initiated only after obtaining approval from the Review Committee of DUHS.

## Results

A total of 573 questionnaires, both manual (300) and online (273), were included in the study. Among the manually distributed questionnaires, 298 were returned with a response rate of 99.3%. Table 1 presents a summary of the first portion of the questionnaire associated with the demographic and socioeconomic aspects.

**Figure 1:**
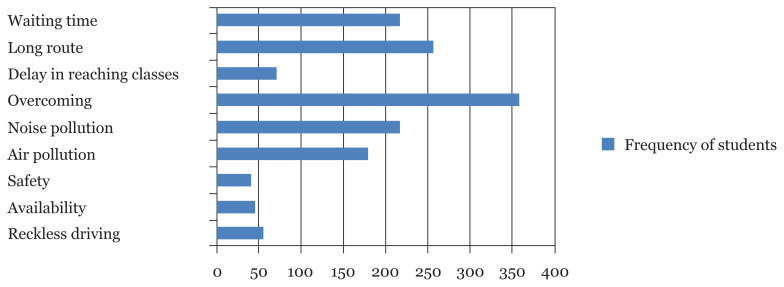
Stress-related factors as contributors of stress

Out of the 573 students, 65.4% used university transport, 3.5% used personal transport driven by self and 14.1% driven by others, 7.7% used online transport services, 7.9% used public transport and 1.4% students came walking. When asked about their ideal means of transport, more than 90% of students selected private transport, either personal or online car services. Students were asked to opt for all stressors in their present means of transport, results of which are summarised in Figure 1.

Figure 1 shows that students revealed multiple contributors to their stress, with the majority of students stating overcrowd, long traveling time and pollution as the main source of their stress and tension.

With more than 90% of students living at a distance of more than 5 km from the institute, traveling time was less than 30 min for 13.1%, between 30 min and 60 min for 30.9%, and more than 1 h for 66%, among which 9.8% had a traveling time of more than 2 h. In addition, 47% of the students reported being extremely stressed by noise and 69.5% believed that transportation consumes excessive time that could be used in fruitful endeavours elsewhere. During commuting, 15.4% of students reported having suffered physical trauma and 31.6% of students had faced physical, verbal or psychological harassment while commuting. The portion of the survey on health revealed that 52.7% of students had an average or below average sleep quality. Moreover, 7.7% of students consumed some drugs, with cigarette smoking being the most common (2.7%). In addition, 74.9% of students reported having fallen ill in the past 1 month, with 50.6% falling ill once or twice, 18.2% a few times and 6.1% very frequently. A total of 489 students (85.2%) reported having suffered from common cold/flu in the past 1 month, whereas 351 students (61.3%) had some illnesses or diseases aggravated in the past 1 month.

Sheldon Cohen’s PSS was used to assess the stress level, wherein only 7.2% of students were reported in the low, 66.5% in the moderate and 26.4% in the high stress categories.

The variables were cross-tabulated using the Chi-square test. A *P*-value of < 0.05 was considered statistically significant. All variables were cross-related to stress levels, including the mode of transport, time duration during transport, noise exposure, sleep quality, ability to meet academic guidelines, trauma (both experiencing and witnessing), harassment, frequency of illness and economic condition. A significant association was found between stress levels and noise exposure (*P* = 0.023) and sleep quality (*P* = 0.001). However, no direct association was reported between the mode of transport opted and stress levels (*P* = 0.1), showing that several other factors play a major part in determining stress in an individual, with transport playing a minimal role. Table 2 shows the results of a univariate analysis between stress and several major factors.

A multivariate regression analysis was performed using the Wald test for quantifying the role of each factor in the overall stress of medical students (Table 3). The logistic regression analysis showed the explanatory factors for stress in medical students. Students who were under stress compared with no stress individuals were females (adjusted odds ratio [AOR] = 1.92), belonged to the third-year class (AOR = 1.55), annoyed by noise (AOR = 1.70) and used cab/taxi or walked to university as a mode of commute (AOR = 1.89). The most strongly associated factor with stress was poor sleep quality, and odds of having poor sleep quality among stressed students were 7.24 compared with that of control participants. The model explained 8.5% (Nagelkerke R^2^) of the variance in stress and correctly classified 75% of the sample.

## Discussion

Psychological stress in medical students has been a leading cause of the increased amount of burnout, fatigue, depression and anxiety (10, 11). The disturbing effects of burnout could vary from low productivity levels, mental and physical exhaustion, strained relationships and suicidal ideation (9). In this study, we studied the prevalence of stress levels and their association with factors related to transportation. A wide range of data collected from both the developing and developed countries has repeatedly established the association between transportation and stress, predominantly affecting medical students at large. This has been evaluated by the effect of traveling on not only medical students but also truck and freight drivers and those whose professions revolve around traveling (15).

The study of Humayun et al. (1) was in line with our study in terms of its setting to evaluate the effect of transportation on female students. To the best of our knowledge, this study has been the first to examine a multivariate model on a large sample of a diverse population in the metropolis of a developing country.

A majority of the students (92.9%) were rated having moderate-to-high levels of stress, which was more statistically in line with the findings of Humayun et al. (48.3%) (1). This can be because of various reasons. First, DMC is situated in the downtown of the city, which is a more industrialised and populated area as compared with Karachi Medical and Dental College (KMDC). Students must travel long distances and encounter heavy traffic daily that may lead to increased stress levels (3). Second, we selected an externally validated scale (PSS) to measure our findings in contrast to the study of Humayun et al. (1), where no such measure was used. This indicated that people generally might have a personal bias, have underestimated, or be hesitant to report accurate results. Furthermore, depressed students seem to have a higher discrepancy in reporting wherein they tend to under-or overestimate the results (16).

Third, DMC has a modular examination system as compared with the annual system of KMDC, indicating that conducting exams throughout the year is a definite contributor toward high stress levels, burnout, anxiety and depression rates (17).

Interestingly, third-year students were considered to be more stressed than their counterparts. This is on par with the belief that students starting their clinical training were more likely to experience increased rates of stress and burnout in the initial year (18). Increased modular and shelf exams and a lesser duration of exam preparation may be the additional reasons. However, students’ perception of a particular stress contributor was not analysed. A similar study (17) showed that the replacement of the annual system with the semester system changes routine activities such as sleep deprivation and increased intake of stimulants, which further aggravated stress.

Our study reported that approximately half of the participants rated their sleep quality to be average or below average, which can be justified by the higher stress levels similar to that noted in previous studies (4, 17). The quality of sleep has been a significant determinant of memory function, cognitive performance and concentration span. Poor sleep quality has been reported to have a deteriorating effect on health as it stimulates the hypothalamus-pituitary-adrenal axis. This pathway leads to increased cortisol levels, as observed by both noise exposure and high stress levels (19). The possible reasons for sleep deprivation are increased caffeine intake (17), increased travel time and academic burden (4).

Noise pollution during commuting was significantly associated with increased stress levels. The relationship of noise pollution with stress and other diseases is also well established on a biochemical basis, leading to elevated levels of stress hormones such as cortisol (19) and dopamine (20). Additional studies in freight and truck drivers have established noise and sleep deprivation to be a cause of increased mental health problems and frustration, leading to traffic accidents (18).

Interestingly, transport and duration were not significantly associated with stress levels, although several students have enlisted some major transport-related stressors. Overcrowding was considered one of the most significant contributors to anxiety and uneasiness, which has been pertinent because of the reasons indicated. Particularly in females, overcrowding was perceived as a greater risk factor, leading to opportunistic harassment (21). However, the pattern of occurrence of experiencing (15.4%) or witnessing trauma (30%) and harassment (31.6%) was somehow less than that noted in the previous study (22). According to our study, this may be because the majority of the students prefer university transport (65.4%) rather than public transport. Public transport has been associated with increased incidences of sexual harassment and events causing psychological or physical trauma. Other studies reported that the prevalence of harassment was approximately three times as compared with our study (22, 23). This study was conducted on women using public transport as their only means of commute as compared with our study where the majority of women used university buses, which may explain the present discrepancy. Notably, the amount of stress was consistently similar across different types of transportation and students who just walk to university experienced significant stress. The multivariate regression analysis revealed that in addition to other factors, female gender and those who walk or take a cab were more likely to be stressed. Particularly, walking or using cab services was slightly a significant (*P* = 0.046) factor in causing stress. This suggests that a female traveling alone, even for a shorter duration, feels unsafe because of the higher prevalence of harassment and criminal events. Furthermore, the high cost of cab services adds to the financial strain.

Students seemed deeply concerned regarding the time consumption because of the long route, waiting time and delay in reaching classes. Although the long route was reported as the most annoying factor, it was found to be insignificant. Students (90%) preferred a personal vehicle driven by others, indicating that the mode of transportation may assist in lowering stress levels. Friman et al. (24) found that active commuting such as cycling assisted in lowering stress levels even for a longer duration. However, in this study, because of the long route, this mode of transportation was not used as other options seemed to be safer and quicker.

Students could meet their deadlines on time, ranging from sometimes (43%) to most of the time (29.0%), which was less when compared with the study of Sohail et al. (9) that examined stress levels and academic performance. This indicated that students still need to develop better coping mechanism as suggested in the earlier published evidence (25).

The socioeconomic condition, although perceived to be a significant stressor, mostly in females (26), was not significant in our study. This lack of significance may be explained by the fact that more than half of the participants belonged to the upper-middle class, and being a public sector university, the fee structure was highly economical when compared with other private sector universities.

A majority of the students (74.9%) in our study reported being recently experiencing mild-to-moderate illness. This was in parallel with Duric et al. (27), concluding that chronic stress has hazardous effects on every major organ system and may lead to an early mortality burden.

To counter stress levels and achieve deadlines on time, both local and international studies have reported the active use of stimulants and drugs among medical students (28, 29). In this study, one in 14 students were admitted for using recreational drugs and stimulants at present. In par with our study, a local study conducted by Imran et al. (29) found tobacco use to be the most frequent form and much higher (35.06% versus 78.9%). Alcohol was considered to be the second most prevalent substance use (26.2%), unlike our study, wherein no such finding was reported. In addition, this contrasted with other studies where the incidence of drug abuse was higher (28). On investigation, living close to or with the families, religious beliefs and cultural taboos (28) were some of the factors that may account for such abstinence in our population.

Our final regression model showed a significant association of stress with gender, noise during commuting, using a cab or walking, being a third-year student and poor sleep quality. Noise pollution and gender were positively associated with stress and sleep quality was negatively associated. The association of poor sleep with stress and stressful conditions is a known fact and has been widely reported (4, 17, 30, 31). A strong association between sleep and stress in our study highlighted the need for countermeasures in medical students. However, an element of reverse causality cannot be reduced here. In addition, in a cosmopolitan city with an estimated population of approximately 20 million, poor transportation infrastructure has a synergistic effect on increasing stress and poor sleep quality. Females were reported to be more stressed than males, which was consistent with the previous studies (11, 18). Possible sociocultural factors included harassment, household responsibilities, peer pressure and poor transportation services. A study stated that 55.4%–60.0% of the females considered transport as the major problem faced by them (23). Males were normally hesitant or shy to report any distressing factor, which may result in underreporting (25).

## Limitations and Strengths

There were several strengths of this study. According to the best of our knowledge, this is the first study to analyse large and diverse cohorts in a public university of a developing country-oriented in the downtown of the city. In addition, we adopted a multifactorial approach covering demographics, transportation-related stressors and all the stressors mentioned in the literature review, which have not been previously examined in much detail. Rather than solely relying on the students’ perception regarding stress, we characterised stress on the basis of a validated stress scale.

Due to its cross-sectional nature, students probably experience different levels of stress, particularly more during the pre-examination era. The total sample included five different batches, each undergoing different phases of the academic schedule. Therefore, perceived stress levels may vary during the year. Moreover, our study results were consistent with that of the previous studies, although coping mechanisms and stress-lowering techniques could only be suggested and were not examined in detail. The association of burnout and stress levels with personal and professional satisfaction was not analysed.

## Future Recommendations

The primary aim of our study was to determine the contribution of transportation to already prevalent stress. Other stressors could likely have somehow outweighed the effect of transport-related stressors. Therefore, it is unlikely that one study may be accurate in confirming or ruling out transportation as a crucial factor. Therefore, multiple longitudinal studies should be conducted to evaluate the long-term effects of stress on academic performance and the health of students. Furthermore, transportation-related stressors need to be studied in much detail. In addition, it will be interesting to explain how stress levels change when these students go into more challenging clinical training and how it affects their views regarding the choice of specialty.

## Figures and Tables

**Table 1:** Sociodemographic and academic characteristics of the selected participants (*n* = 573)

Factors	Frequency (*n* = 573)	Percentage
Gender		
Male	108	18.8
Female	465	81.2
Year of study		
1st	126	22
2nd	141	24.6
3rd	196	34.1
4th	57	9.9
5th	54	9.4
Residence with		
Family	548	95.5
Without family	25	4.5
Socioeconomic status		
Lower class	5	0.9
Lower-middle class	69	12
Upper-middle class	319	55.6
Upper class	180	31.5
Employment status		
Employed	117	20.4
Unemployed	456	79.6

**Table 2:** Association of transportation-related factors with stress levels among student participants (*n* = 573)

Variables*	Low stress (%)	High stress (%)	Odds ratio	95% Confidence interval (rounded off to 2 decimal point)	*P*-value
Gender					
Male	82.4	17.6	0.54	0.32 0.92	0.022
Female	71.6	28.4			
Year of study**					0.018¶
1st ∞	69.8	30.2	–		
2nd	76.6	23.4	0.71	0.41 1.22	0.212
3rd	78.6	21.4	0.63	0.38 1.05	0.077
4th	57.1	42.9	1.74	0.91 3.33	0.095
5th	74.1	25.9	0.81	0.40 1.66	0.566
Noise annoyance**					
Not at all∞	82.4	17.6	–	–	0.023¶
A little	73.9	26.1	1.65	0.61 4.46	0.323
Moderately	74.2	25.8	1.63	0.63 4.18	0.309
A lot	77.1	22.9	1.39	0.53 3.59	0.501
Extremely	64.1	35.9	2.62	0.99 6.90	0.046
Sleep quality**					
Very poor∞	33.3	66.7	–	–	< 0.001¶
Poor	65.2	34.8	0.27	0.10 0.75	0.010
Average	73.6	26.4	0.18	0.07 0.47	< 0.001
Good	79.7	20.3	0.13	0.05 0.34	< 0.001
Near perfect	77.7	22.3	0.14	0.05 0.40	< 0.001

Notes:

* Chi-square test was used to evaluate the categorical variables;

** Odds ratio of the variables (year of study, noise annoyance and sleep quality) has been found by comparing other years against the first year, higher annoyance levels against not at all, and a better degree of sleep quality against very poor, respectively;

¶ *P*-value from Chi-square test showing an overall difference among multiple categories;

∞ Reference category for the respective variable

**Table 3:** Multivariable logistic regression analysis showing risk factors for stress among university students (*n* = 573)

Characteristics of students	B	SE	Wald	df	Sig.	AOR	95% Confidence interval for AOR	

							Lower	Upper
Female gender	0.65	0.28	5.32	1	0.021	1.92	1.10	3.35
3rd year student	0.44	0.22	4.15	1	0.042	1.55	1.02	2.37
Annoyed by noise	0.52	0.24	4.82	1	0.028	1.70	1.06	2.69
Poor sleep quality	1.98	0.49	16.34	1	< 0.001	7.24	2.80	18.91
Walk or use cab/taxi	0.63	0.32	3.98	1	0.046	1.89	1.01	3.50

Note:

* The multivariate regression analysis has been performed using the Wald test
